# Targeting lipid metabolism in the treatment of ovarian cancer

**DOI:** 10.18632/oncotarget.28241

**Published:** 2022-05-25

**Authors:** Saliha Chaudhry, Stefani N. Thomas, Glenn E. Simmons Jr.

**Affiliations:** ^1^Department of Medicine, University of Minnesota School of Medicine, Twin Cities, MN 55455, USA; ^2^Department of Laboratory Medicine and Pathology, University of Minnesota School of Medicine, Twin Cities, MN 55455, USA; ^3^Department of Biomedical Sciences, Cornell University College of Veterinary Medicine, Ithaca, NY 14853, USA

**Keywords:** ovarian cancer, lipid metabolism, biomarkers, microenvironment, fatty acid

## Abstract

Cancer cells undergo alterations in lipid metabolism to support their high energy needs, tumorigenesis and evade an anti-tumor immune response. Alterations in fatty acid production are controlled by multiple enzymes, chiefly Acetyl CoA Carboxylase, ATP-Citrate Lyase, Fatty Acid Synthase, and Stearoyl CoA Desaturase 1. Ovarian cancer (OC) is a common gynecological malignancy with a high rate of aggressive carcinoma progression and drug resistance. The accumulation of unsaturated fatty acids in ovarian cancer supports cell growth, increased cancer cell migration, and worse patient outcomes. Ovarian cancer cells also expand their lipid stores via increased uptake of lipids using fatty acid translocases, fatty acid-binding proteins, and low-density lipoprotein receptors. Furthermore, increased lipogenesis and lipid uptake promote chemotherapy resistance and dampen the adaptive immune response needed to eliminate tumors. In this review, we discuss the role of lipid synthesis and metabolism in driving tumorigenesis and drug resistance in ovarian cancer conferring poor prognosis and outcomes in patients. We also cover some aspects of how lipids fuel ovarian cancer stem cells, and how these metabolic alterations in intracellular lipid content could potentially serve as biomarkers of ovarian cancer.

## INTRODUCTION

Ovarian cancer is a lethal and common gynecological malignancy, with 80% of patients being diagnosed at an advanced stage of disease [1]. Fewer than half of patients survive beyond five years after diagnosis due to the prevalence of aggressive high-grade serous carcinomas and lack of accurate early detection methods. As with many other cancers, much of what we know about ovarian cancer relates to genetic abnormalities that give cell growth or survival advantages. Mutations in genes such as TP53, PTEN, KRAS, and Rb1 are considered major driver mutations in many cancers and are in part responsible for establishing ovarian tumors [2–4]. However, some of these mutations, such as KRAS, are conspicuously absent in the most lethal form of OC, high grade serous ovarian cancer (HGSOC). A possible explanation for this observation is the presence of other cancer-specific adaptations that are independent of DNA mutation.

Research has shown that increased lipid uptake supports the high energy needs of growing malignant cells, with alterations in lipid metabolic genes often already present in early stages of ovarian cancer and becoming more prevalent with disease progression [5]. Lipid metabolism is critical to the growth and proliferation of all eukaryotic cells. Lipid metabolism refers to the synthesis, catabolism, and uptake of lipids from the surrounding environment. The metabolism of lipid molecules is important for normal cell biology and is critical to the development of pathological conditions, such as cancer.

Lipids have a dynamic role in the context of tumorigenesis in the ovaries, and they are involved in supporting cancer cell growth and suppressing the immune response. Lipid intermediates in ascites and fat-containing cells of the omentum have been shown to negatively affect the function of T-lymphocytes, which could inhibit the anti-tumor activity of the immune system [6–9]. Additionally, cancer cell survival and spread has been linked to increased levels of lipogenic enzymes. For instance, fatty acid synthase (FASN) and stearoyl-CoA desaturase (SCD1) are elevated in high-grade, metastatic ovarian tumors and lead to elevated levels of unsaturated fatty acids [5, 10, 11]. This increase in lipogenic enzymes, and their products (e.g., unsaturated fatty acid) often correlates with poor patient outcomes. Therefore, it is of interest to examine the impact of alterations in lipid metabolism to gain a better understanding of ways to subvert the molecular pathogenesis seen in ovarian cancer. The current review presents a logical argument for developing more approaches to therapeutically target the lipogenic pathways in cancer cells to improve patient outcomes.

## LIPID DYSREGULATION IN CANCER

**Figure 1:**
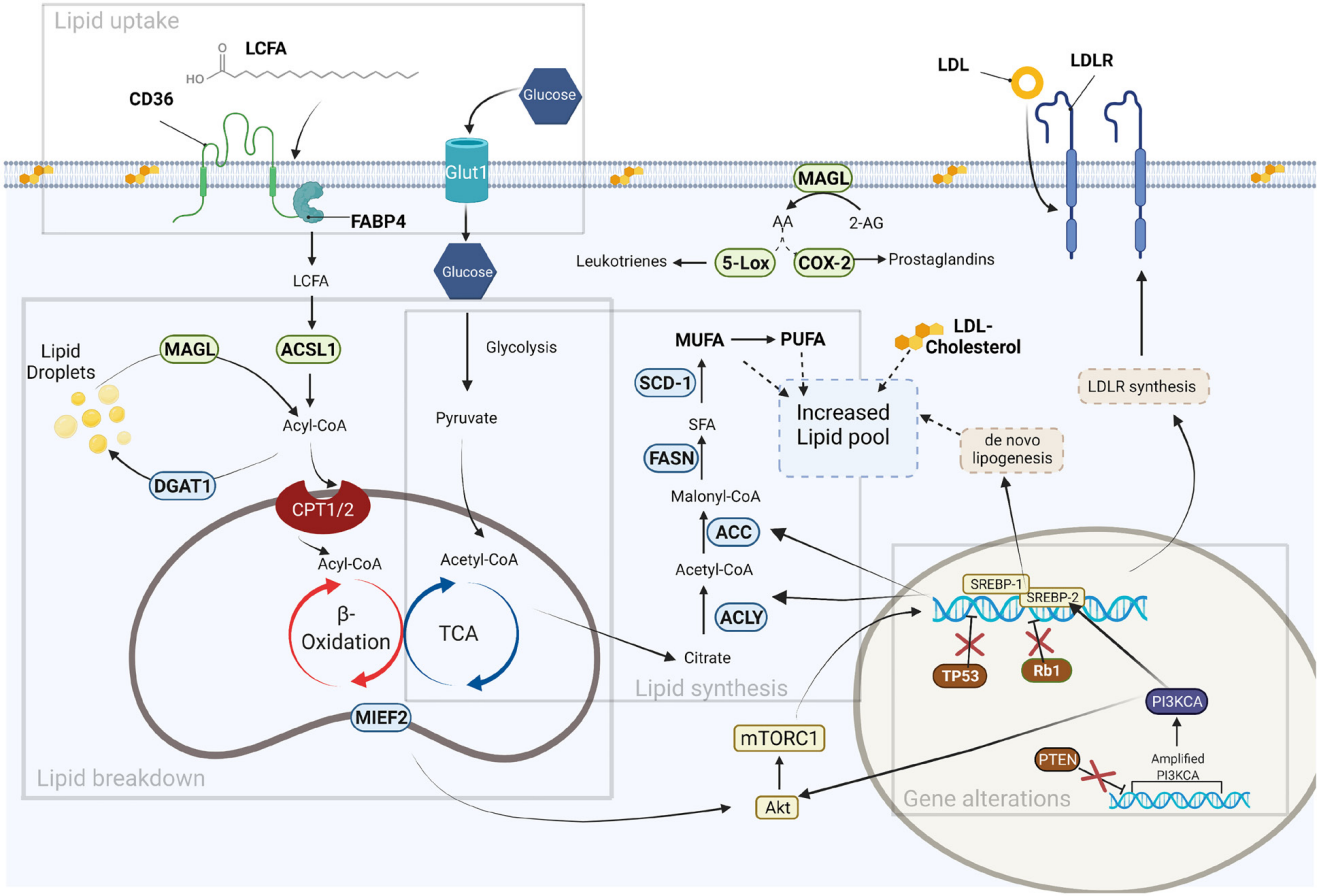
Fatty acid metabolism in cancer. Gene alterations: Several gene alterations (mutations, amplifications, deletions) contribute to increased production of lipogenic genes either directly via transcription regulation, or indirectly by loss of repressors. Lipid uptake: Fatty acids (FAs) are obtained via de novo lipogenesis and exogenous uptake. FA translocase CD36 is responsible for the exogenous uptake of FAs from the surrounding microenvironment. These FAs can be converted to triacylglycerols (DGAT1) and stored in lipid droplets or used in generation of acetyl-CoA through β-oxidation. Lipid Synthesis: Glucose is a major carbon source for *de novo* lipogenesis. Pyruvate derived from glucose contributes the substrate for several lipogenic enzymes (ACLY, ACC, FASN, SCD-1) leading to an increased lipid pool. Lipid breakdown: Lipid droplets are mobilized by lipase enzymes (MAGL) to provide energy for cancer cell growth and secondary bioactive lipids that modify the tumor microenvironment. Several promising lipid-targeting anti-cancer therapies are based on disrupting the lipid metabolic pathways (enzymes, receptors, and bioactive lipids) that are shown in this figure. Created with https://biorender.com.

As stated earlier, lipids play important roles in biological processes in eukaryotic cells. Abnormal lipid homeostasis is pathognomonic with several diseases such as metabolic syndrome, obesity, diabetes, liver steatosis, cardiovascular disease, and cancer. Rapidly dividing cancer cells produce significantly more fatty acids and sterols (for energy and increased membrane synthesis) compared to non-transformed cells. In addition, cancer cells derive nearly 95% of their saturated and mono-unsaturated fatty acids *de novo*, even in the presence of adequate dietary lipids. Furthermore, studies have suggested that cancer cells also utilize lipolysis (the breakdown of fatty acid) to provide additional raw materials for cellular energetic demands [12–14]. This combination of metabolic alterations has led some researchers to suggest that tumorigenesis is the consequence of epithelial cells capitalizing on an overabundance of lipids in the environment (Figure 1) [15, 16].

In transformed cells, lipid metabolism, along with several other biosynthetic pathways, is increased to keep pace with the energetic demands of the rapidly proliferating cancer cells. For many years, researchers focused almost exclusively on glucose metabolism, but more recently other aspects of metabolism have come into the spotlight as well, including amino acid and lipid metabolism [17, 18]. Cells with high metabolic demands often experience alterations in key genes leading to increased expression of genes related to lipid synthesis [14, 19–21]. Interestingly, genes related to lipogenesis are not often mutated in cancer, leading many to believe that lipid alterations in cancer are a compensatory effect in response to other driver mutations. In support of this idea, many cancers, including OC, are known to possess “driver mutations” in genes that regulate the expression and activity of many lipogenic genes to the benefit of growing cells [22]. For instance, major driver mutations associated with ovarian carcinoma development are in genes encoding for PTEN, TP53, and Rb1, [4]. Mutations in these genes lie upstream of several highly regulated enzymes involved in various pathways that converge on lipid metabolism.

## MUTATIONS IN GENES THAT AFFECT LIPOGENIC ENZYMES INVOLVED IN OVARIAN CANCER

As therapeutic interests in lipid metabolism of cancer have increased, an increasing number of studies have highlighted how some of these enzymes are involved in affecting the characteristics of ovarian tumors. As mentioned earlier, alterations in lipid metabolism appear to stem from effects of mutations in genes upstream of the lipogenic pathway. However, these mutations substantially impact the overall metabolic conditions within the cell and provide a boost to lipid production and turnover. What begins to come clear is that the presence of excess lipids provides growth advantages to transformed cells that should be considered when developing therapeutic approaches.

### PTEN

Phosphatase and tensin (PTEN) homolog is a multi-functional tumor suppressor found in nearly every cell of the body. Its deletion on Chromosome 10 is associated with oncogenesis. As a tumor suppressor, PTEN functions as a negative regulator of another enzyme associated with oncogenesis, PI3KCA [23]. PI3KCA is rarely mutated in serous OC, but the PI3KCA gene is often amplified leading to increased activation of Akt and mTORC1 signaling (a major control center for cellular metabolism) and activation of sterol response element binding proteins (SREBP1 and SREBP2) [24–27]. SREBP1 is the master regulator of lipid biosynthesis and controls the expression levels of many biosynthetic genes such as fatty acid synthase (FASN), SREBP2, acetyl CoA carboxylase (ACC), ATP citrate lyase (ACLY), HMG-CoA carboxylase (HMGC), HMG-CoA reductase (HMGCR), low density lipoprotein receptor (LDLR), and many others [24]. Taken together, loss of PTEN via gene deletion in cancer cells can explain a fair amount of increased lipogenesis.

### TP53

In addition to the tumor suppressor PTEN, Tumor protein 53 (TP53) is often downregulated in several models of cancer [2, 28, 29]. TP53 mutations are almost ubiquitous in HGSOC, and yet deletion of TP53 alone does not lead to the transformation of ovarian surface epithelial cells. This strongly suggests that p53 is not a unique driver of tumorigenesis but instead works in concert with other mutations [4]. This idea is supported by evidence in HGSOC tumors with TP53 mutations, roughly 67% of which also contain mutations in the Rb1 gene. In models of ovarian cancer, it has been estimated that 5–8 genetic mutations are needed to establish oncogenic growth. This suggests that loss of functional TP53 and Rb1 synergize in HGSOC to promote tumorigenesis.

### RB1

Retinoblastoma protein (Rb1) is a cell cycle checkpoint regulator that controls the cell’s transition from G1 to S-phase. In cancer development Rb1 is considered a tumor suppressor and therefore its deletion is closely correlated with the development of several malignancies, in particular retinoblastoma [30–32]. Rb1 is involved in several metabolic pathways including autophagy, glycolysis, oxidative phosphorylation, and mitochondrial biogenesis [31]. Studies investigating the cellular role of Rb1 show that Rb1 deletion leads to increased production of lipids, especially fatty acids, as cells enter the cell cycle. This lipogenic effect is mediated by increased binding of the E2F transcription factor to the promoter of target genes like SREBP1. Increased expression and activity of SREBP1 leads to increased expression of stearoyl-CoA desaturase (SCD1) and fatty acid elongase 6 (ELOVL6). The activity of SCD1, and to a lesser extent ELOVL6 are responsible for the increased levels of unsaturated fatty acids present in cancer cells [30].

## LIPOGENIC ENZYMES DYSREGULATED IN OVARIAN CANCER

### Stearoyl CoA desaturase (SCD1)

It is important to understand that increased lipogenesis and uptake can be potentially toxic to cells. When cellular lipid stores decline, they signal the activation of the transcription factor SREBP1. This leads to corresponding increases in the expression of fatty acid synthetic genes including FASN, ACC, ACLY, and LDLR. The production of fatty acids potentiates the transformation of acetyl-CoA into saturated fatty acids, palmitate, and stearate. However, high levels of saturated fat are detrimental to membrane integrity and are therefore toxic to cells This is called lipotoxicity [33, 34]. Lipotoxicity occurs when fatty acid or sterol accumulation exceeds the ability of the cell to package them into triacylglycerols and sterol esters [35]. In cancer cells, as fatty acid synthesis increases, the conversion of saturated fat to unsaturated fatty acids also increases due to the activity of stearoyl-CoA desaturase (SCD1) [36].

SCD1 is a delta-9 desaturase that introduces a single double bond into the 9th position of stearic and palmitic acid (making oleic and palmitoyl oleic acid, respectively) [37]. SCD1-mediated production of unsaturated fatty acids is essential to produce cell membranes and phospholipids as unsaturated lipids are required for proper membrane function [38, 39]. Many studies have demonstrated that SCD1 levels are elevated in cancer compared to normal cells [40–42]. Accordingly, SCD1 has been proposed as a therapeutic target [11, 38, 43–47]. In preclinical studies, pharmacological inhibition of SCD1 blocks cell proliferation when exogenous lipids are limited [48]. This suggests that cancer cell dependence on lipids needs to be thwarted both at the synthesis and uptake stages to have an integrative anti-cancer effect. Although clinical data in this regard is still limited, there is evidence that populations of ovarian cancer stem cells are heavily reliant on SCD1 activity [11].

Cancer stem cells are regarded as being responsible for much of the proliferative capacity in tumors as well as resistance to therapy. In single cell analysis, lipid desaturation markers were elevated in cancer stem cells when compared to non-stem cancer cells [11]. Furthermore, analyses of cell proliferation and migratory capacity were shown to be largely supported by the presence of unsaturated fatty acids. Taken together, it appears that the presence of unsaturated fatty acid contributes significantly to the functions of cancer cells that are associated with poor disease outcomes.

### ATP-Citrate lyase (ACLY)

It is not only the latter steps in fatty acid generation that are altered in OC. Key initiating enzymes are also altered to some extent as well. ATP-Citrate Lyase (ACLY), an upstream regulator of fatty acid synthesis, is responsible for converting 6-carbon molecules (glucose and glutamine) into molecules of oxaloacetate and acetyl-CoA. ACLY links sugar- or glycol-metabolism to lipid metabolism, illustrating the important relationship between elevated glucose uptake and lipid metabolism in cancer cells [49]. Increased ACLY levels are beneficial to OC cell fitness [2]. Correspondingly, targeting ACLY with interfering RNAs decreases the proliferative capacity of ovarian cancer cells, demonstrating its critical role in supporting cancer cell growth [50].

### Acetyl CoA carboxylase (ACC)

Continuing along the fatty acid synthetic pathway, Acetyl-CoA is similarly elevated in OC. Acetyl-CoA Carboxylase is the enzyme responsible for the first committed step in fatty acid synthesis, catalyzing the carboxylation of cytosolic acetyl-CoA to form malonyl-CoA. ACC activity is regulated during post-translational phosphorylation by adenosine monophosphate kinase (AMPK). In ovarian cancer, inhibition of ACC with the allosteric inhibitor TOFA induces G0/G1 cell cycle arrest and apoptosis [51]. AMPK-ACC pathways in cancer cells are regulated by lysophosphatidic acid (LPA), a bioactive lipid-like growth factor mediator, which is found in ascites of ovarian cancer patients at high levels. Increased activity of ACC, along with fatty acid synthase, drives *de novo* lipid production. Studies examining LPA-mediated mechanisms in ovarian cancer reveal the role of LPA in activating AMPK-ACC cascades, resulting in an increase of *de novo* lipogenesis [52]. There is even evidence that increased ACC activity in response to upstream metabolic signals (increased AMPK activity) is partly responsible for the resistance of ovarian cancer cells to chemotherapy [53].

### Fatty acid synthase (FASN)

In support of the central role lipid production plays in cancer cell survival, inhibition of SREBP1 has been demonstrated to prevent the growth of ovarian cancer in xenograft models [26]. In a more focused analysis, targeting the final enzyme of the canonical fatty acid biosynthetic pathway, fatty acid synthase (FASN), showed very similar results. FASN is essential for lipid synthesis and uses acetyl CoA derived from glucose to synthesize palmitate and other fatty acids used in lipid signaling, cell proliferation, and triglyceride storage [54]. Early studies that monitored FASN expression in primary prostate cancers showed that FASN expression was detected in 57% of 99 primary prostate cancers, which corresponded to decreased disease-free survival in patients [55]. This is corroborated by another study that showed FASN functions as an oncogene when expressed in excessive amounts [56]. Increased levels of FASN have been linked with decreased patient survival, increased disease recurrence, and increased invasive capacity of cancer in patients [19, 57].

More recent work suggests that FASN expression is associated with worse outcomes in cancers, including ovarian cancer. [55, 56, 58, 59]. This observation is likely linked to the association of increased FASN with resistance to cytotoxic stress induced by chemo- radiotherapy, leading to poorer patient outcomes. Several studies have demonstrated that inhibition of FASN prevents the growth of the immunosuppressive phenotype associated with a variety of cancers including ovarian cancer, further implicating its critical role in tumorigenesis [10, 17, 60–62]. Inhibitors of FASN have demonstrated efficacy as anticancer therapies based on the results of studies using cell lines and ovarian tumor mouse xenograft preclinical models [50, 51, 63].

### Mitochondrial elongation factor 2 (MIEF2)

Even outside of the canonical lipogenic/ fatty acid biosynthetic pathway there are enzymes whose activities affect lipogenic properties of cancer cells. One such molecule is Mitochondrial elongation factor (MIEF2), a regulator of mitochondrial fission. In the context of ovarian cancer, high expression of this protein is predictive of a poor prognosis. It has been shown that knockdown of MIEF2 decreases levels of free fatty acid, triglycerides, and cholesterol in ovarian cancer cell lines; the converse has also been observed [64]. The increase in lipogenic genes caused by increased expression of MIEF2 appears to be the result of increased activation of the ROS/Akt/mTOR pathway, which results in increased SREBP1 and SREBP2 mRNA levels. As discussed earlier, increased expression of SREBP1 ultimately results in increased levels of lipids and preferred growth conditions in the affected cells.

### Diacylglycerol O-Acyltransferase 1 (DGAT1)

As cells generate increasing levels of lipid molecules even as they are converted to less problematic unsaturated species, they still must be exported or stored. Storage comes in the form of di- and triacylglycerols. Along with SCD1, the protein Diacylglycerol O-Acyltransferase 1 (DGAT1) helps with the toxification of lipid. DGAT1 is responsible for catalyzing the conversion of diacylglycerol and fatty acyl-CoA to triacylglycerol. DGAT1 is overexpressed in ovarian cancer and correlates with poor survival of patients. In fact, the levels of DGAT1 are positively associated with ovarian tumor growth [65]; the larger the tumor, the more DGAT1 will be produced, likely supporting the storage of ever-increasing amounts of lipid. Due to its role in aiding lipid storage, DGAT1 activity is regulated in part by the availability of glucose or glycolytic activity of the cell, as glycolysis provides the raw material that will eventually become new acyl-CoA molecules.

## ENZYMES INVOLVED IN LIPID DEGRADATION IN OVARIAN CANCER

Not only is the ability to generate large amounts of lipid a benefit to cancer cells, breakdown and conversion of lipid is also critical to tumor development and growth. Several studies have shown that enzymes involved in lipid degradation are elevated in cancer cells when compared to non-transformed cells [22, 64, 66, 67]. In some cases, enzymes can be involved in both the biosynthesis and degeneration of lipids such as ACSL1 [67].

### Acyl-CoA synthetase long chain family member 1 (ACSL1)

Acyl-CoA Synthetase Long Chain family member 1 is an isozyme of the long chain fatty acid coenzyme A ligase family. ACSL1 converts free long chain fatty acids into fatty acyl CoA esters and plays a key role in lipid biosynthesis and fatty acid degradation during beta-oxidation. Highly metastatic ovarian cancer cells have a distinct lipid profile as compared to less metastatic cells [66]. There is a notable increase in phospholipids, in particular phosphatidylcholine in these cells. ACSL1 overexpression in non-metastatic ovarian cancer cells increases their metastatic dissemination in xenograft models, indicating that ACSL1 activity can drive metastasis.

### Arachidonate 5-Lipoxygenase (5-LOX)

Arachidonate 5-Lipoxygenase (5-LOX) is an enzyme responsible for catalyzing the synthesis of bioactive, proinflammatory lipids known as leukotrienes from the polyunsaturated fatty acid arachidonic acid. 5-LOX is highly expressed in ovarian cancer cells and correlates with poor prognosis in patients [5]. 5-LOX-derived leukotrienes are associated with increased cell migration and metalloproteinase expression, leading to increased metastasis [68]. 5-LOX also promotes recruitment of pro-inflammatory tumor associated macrophages (TAMs) into hypoxic regions of the tumor. Increased TAMs density within the tumor is associated with metastasis and tumor stage. Furthermore, it was determined that metabolites of 5-LOX helped promote a positive feedback loop in which the hypoxic environment helped recruit more TAMs, which in turn promoted 5-LOX activity. Similarly, expression of leukotriene receptors (Leukotriene B4 receptor B2) has also been demonstrated to correlate with poor clinical outcomes in ovarian cancer patients [69]. The presence of inflammatory leukotrienes driven by the expression of 5-LOX and its receptors may be a treatment target worth further investigation in ovarian cancer patients.

### Cyclooxygenase-2 (COX-2)

Cyclooxygenase-2 is another rate-limiting enzyme involved in the metabolism of the polyunsaturated fatty acid, arachidonic acid, into bioactive prostaglandins. In ovarian cancer, COX-2 expression is increased leading to increased presence of its product, the Prostaglandin E2 (PGE2) [5, 70]. The synergistic presence of COX2 and PGE2 has been implicated in promoting the expression of the pro-angiogenic cytokine vascular endothelial growth factor (VEGF) [5, 68]. Several studies have demonstrated that COX2 expression also promotes metastasis by increasing the expression of metalloproteinase enzymes that degrade the extracellular matrix surrounding tumor cells [71]. There is evidence that celecoxib, a selective inhibitor of COX-2, successfully inhibits growth and induces apoptosis in ovarian cancer cells [72].

### Carnitine palmitoyl transferase

Carnitine Palmitoyl Transferase (CPT) is responsible for helping cells adapt to low glucose conditions by switching to beta oxidation [73, 74]. CPT helps convert long chain fatty acids in the cell into acyl chains that are then subjected to beta oxidation in the mitochondria. CPT expression is high in ovarian cancer, which facilitates the use of the beta oxidation pathway in these cells, simultaneously with glycolysis [75]. This gives ovarian cancer cells a significant growth and proliferation advantage. The effect of the genetic ablation of CPT1 dramatically alters beta oxidation and induces cell cycle arrest and p21-mediated apoptosis, demonstrating the importance of CPT1 to cancer survival [75].

### Monoacylglycerol lipase (MAGL)

Monoacylglycerol lipase (MAGL) is responsible for catalyzing the decomposition of monoacylglycerol into free fatty acids and glycerol. This effectively increases the level of fatty acid within the cell. The expression of MAGL is elevated in ovarian cancer [5]. Elevated MAGL is associated with increased epithelial-mesenchymal-transition (EMT) potential in cancer cells [76].

## LIPID UPTAKE IN CANCER

### CD36

The uptake of lipids from the circulation is a complementary means by which cells maintain internal stores of fatty acids and sterols. An essential protein involved in the uptake of lipids in cells is the scavenger receptor, or CD36, a fatty acid translocase [77]. CD36 is an 88-kDa transmembrane glycoprotein expressed in numerous cell types including macrophages, endothelial cells, and adipocytes. CD36 translocates from the cytoplasm to the plasma membrane, where it binds to low density lipoproteins and transports them across the membrane, which is the functional mechanism whereby CD36 contributes to the total lipid content of cells. Recent studies have shown that CD36 expression is increased in solid tumors of the breast, stomach, and ovary [7, 78, 79]. Increased CD36 expression levels likely occur in response to the increased energy demands on the cell, similar to what occurs in other metabolic pathways.

CD36 plays a role in the initiation of cancer and is correlated with poor prognosis in melanoma and breast cancer [77]. Inhibition and knockdown of CD36 have deleterious effects on cellular proliferation in many cancers, rendering CD36 a diagnostic biomarker and a potential target for therapy. The reason for this potent anti-cancer effect in CD36 inhibition is linked to inactivation of Wnt/Beta-catenin, a major driver of oncogenic cell growth in cancer cells [80]. If CD36 levels are reduced, then the growth signals provided by the Wnt pathway are substantially inhibited as well.

### Fatty acid binding protein 4

Fatty acid binding protein 4 (FABP4) helps to promote the uptake of long chain fatty acids into cells [5, 81]. FABP4 overexpression has been reported in many cancers including ovarian cancer. FABP4 has the potential to predict the presence of residual disease in ovarian cancer [2]. Immunohistochemical-based FABP4 expression appears to be enriched in areas along the carcinoma cell/adipocyte junction, likely owing to its role in transporting lipids into cells that require additional forms of energy [82]. Cancer cell dependence on readily accessible sources of fatty acids is associated with FABP4 becoming an attractive therapeutic target. Studies targeting FABP4 have shown that FABP4-inhibited cancer cells have decreased aggressiveness (less metastasis) and have an increased sensitivity to carboplatin therapy [8, 81].

### Low density lipoprotein receptor (LDLR)

Low density lipoprotein receptor (LDLR) is a membrane protein that transports cholesterol into cells in the periphery, away from the liver [83]. LDLR is an SREBP responsive-gene, and its expression is increased along with many of the lipogenic genes. SREBP2 and LDLR expression is often elevated in chemoresistant cells in ovarian cancer [84]. Accordingly, elevated LDLR expression in ovarian cancer is correlated with a poor response to platinum-based drugs. Conversely, knockdown of LDLR increases sensitivity to platinum-based therapies [85].

## LIPID METABOLISM IN OVARIAN CANCER STEM CELLS

Tumor relapse and resistance to conventional chemo- and radiotherapies leading to fatal metastatic disease has been associated with the development of cancer stem cells (CSCs) [86]. CSCs play a role in treatment failures and cancer progression through their interaction with other cells and molecules in the tumor microenvironment [87]. The importance of the microenvironment can be seen in the interaction between ovarian cancer cells and their stroma through the regulation of ascitic fluid contents in stromal cells and growth processes. Within the microenvironment, cancer-associated fibroblasts, a major cell population in the stroma, have been found to enhance the generation of ovarian CSCs and cause angiogenesis by inducing the secretion of vascular endothelial growth factors (VEGF) [11, 88].

CSCs also accumulate excess lipids and cholesterol inside lipid droplets [11]. Cancer cells, like adipocytes, store excess energy in lipid droplets that can be broken down into free fatty acids. This formation occurs by lipogenic enzymes which are highly expressed in cancer cells and include acetyl-CoA carboxylase, fatty acid synthase, and ATP citrate lyase.

Tumor growth is dependent on active angiogenesis, therefore, vascular reduction results in tumor inhibition in non-adipose tissues [89]. Analysis of Raman scattered imaging and mass spectrometry of lipids shows significant increases in unsaturated lipid levels within ovarian cancer stem cells compared to non-stem cells [11]. Therefore, this provides evidence that metabolic alterations in lipogenesis with the use of increased unsaturated lipids could be a metabolic marker for ovarian cancer stem cells. These alterations in intracellular lipid content develop from the utilization of extracellular lipids or by *de novo* synthesis.

Ovarian cancer stem cells also can differentiate into other cell types which can further contribute to angiogenesis, progression, and metastasis [84]. *De novo* adipocytes in adipose and tumor tissue differentiate from mesenchymal stem cells and can be influenced by external factors from the tumor microenvironment to enable the storage of excessive levels of lipid content. The formation of remodeled adipocytes, due to cancer cell invasion, results in tumor tissue growth by engulfment of adipocyte clusters [89].

## ROLE OF LIPID METABOLISM IN ANTI-CANCER DRUG RESISTANCE

Ovarian cancer is among the diseases wherein excess adiposity has a causal role [90, 91]. Results from a recent large-scale study conducted by Si et al. to identify the risk factors associated with ovarian cancer indicated that body mass index (BMI), body fat percentage, body fat mass, and basal metabolic rate are significantly associated with ovarian cancer [92]. Based on data from a cohort of 13,222 women diagnosed with ovarian cancer in the United Kingdom with >20 years’ follow-up for ovarian cancer incidence and cause-specific mortality, BMI was identified as a modifiable means of improving survival [93].

One way that BMI can be modified is by adhering to a ketogenic diet consisting of low levels of carbohydrates and high levels of fat to create a metabolic state of ketosis. With ketogenic diets, by increasing insulin sensitivity and restricting carbohydrate intake, adipose tissue is selectively decreased while maintaining lean mass [94]. A study of 30 women with polycystic ovary syndrome demonstrated that adherence to a ketogenic diet resulted in reduced central obesity [95]. Consumption of low levels of carbohydrates resulted in preferential loss of fat mass from metabolically harmful adipose deposits, whereas a diet with high levels of carbohydrates resulted in a re-partitioning of lean mass to fat mass. Supporting the effectiveness of diet modification strategies in improving outcomes for women with ovarian cancer, results from the Women’s Health Initiative Randomized Controlled Dietary Modification Trial indicated that a low-fat diet may be associated with beneficial health outcomes [96].

Obesity has a causal role in the anti-cancer drug response of patients with ovarian cancer. More specifically, adipocytes and lipid metabolism play complex roles in modulating anti-cancer drug resistance, which is one of the most significant challenges to successful ovarian cancer treatment. Although the dependence of cancer cells on glycolysis for energy production has been studied extensively [97], less is known about the roles of adipocytes and alterations in lipid metabolic programming in the context of the growth, metastasis, and drug responses of cancer cells. Adipocytes promote ovarian cancer metastasis and provide energy for rapid tumor growth [98]. Adipocyte-rich environments support tumor growth via several mechanisms. In ovarian cancer, metastatic cells are home to omental adipose tissue which contain high concentrations of triglycerides. Free fatty acids are generated from the hydrolysis of these free fatty acids which metastatic ovarian cancer cells uptake and utilize as energy sources. Adipocytes secrete factors that increase ovarian cancer cell resistance against chemotherapeutic drugs by Akt pathway activation, and the Akt pathway has been demonstrated to mediate the anti-apoptotic activity of adipocytes [53]. Additionally, arachidonic acid, a polyunsaturated fatty acid present in cell membrane phospholipids, is capable of activating Akt and inhibiting cisplatin-induced apoptosis [89]. The level of Akt activation is positively correlated with the chemo-protective effect of arachidonic acid.

Among the potential mechanisms that underlie poor anti-cancer drug response in obese cancer patients are adipose hypoxia, altered pharmacokinetics, increased ATP production, altered microbiota, the production of tumor-promoting growth factors and cytokines, and the generation of drug-resistant cancer stem cells [89, 99]. The low-grade hypoxia which occurs in adipose tissues stimulates angiogenesis, inhibits macrophage migration and pre-adipocyte differentiation, increases fibrosis, and suppresses immune cell recruitment [100]. Based on the results from studies using pancreatic cancer cells, Harbuzariu et al. proposed that elevated levels of leptin in obese individuals could protect cells from chemotherapy-induced apoptosis [101]. Obesity could enhance fibrosis in tumors by facilitating interactions between pro-inflammatory and fibrotic pathways, consequently impeding tumor drug delivery [102].

Lipids and cholesterol have critical roles in the proliferation of cancer cells. In these highly proliferative cells, lipid catabolism occurs via the fatty acid ß-oxidation (FAO) pathway, which involves exogenous and endogenous fatty acids [22]. In nutrient- and oxygen-depleted environmental conditions, cancer cells exhibit an increased dependence on FAO [103]. It therefore follows that therapeutic strategies intended to modulate lipid-associated metabolism in cancer cells must be designed to avoid adverse effects on normal metabolic functions.

Ovarian cancer cells utilize lipid metabolism in the ascites or omental microenvironment during metastatic progression through AMPK/ACC/FASN-mediated lipogenesis and AMPK/TAK1/NF-κB signaling pathways. Chen et al. demonstrated that targeting lipid metabolism and/or suppressing TAK1/NF-κB signaling is an effective therapeutic strategy to prevent and treat peritoneal metastases in ovarian cancer cells [104, 105]. Cholesterol-lowering drugs such as statins target lipid metabolism. Statins have been shown to induce the apoptosis of ovarian cancer cells [106]. Lovastatin was utilized in a clinical trial in combination with the chemotherapeutic agent Paclitaxel to improve the standard-of-care for patients with refractory or relapsed ovarian cancer [107]. Another targeted strategy for altering lipid metabolism in ovarian cancer is the knockdown of ceramide transport protein (CERT) [108]. Knockdown of CERT causes the accumulation of ceramide in the ER, increasing ER stress, and sensitizing ovarian cancer cells to Paclitaxel treatment [105].

## LIPIDS AS DIAGNOSTIC OR PROGNOSTIC BIOMARKERS

Analytical methods including imaging techniques and mass spectrometry have enabled the determination of fatty acid composition and intra-tumor lipid class spatial distribution toward the development of lipid and lipid metabolism-associated biomarkers [109, 110]. For example, breast tumors and epithelial ovarian cancer cells have been shown to exhibit increased levels of membrane-associated phosphatidylcholine and phosphatidylethanolamine [111–113]. These phospholipids have potential roles in improving clinical diagnosis as well as the identification of new therapeutic targets.

Promising lipid-targeting anti-cancer therapies are based on disrupting lipid metabolic pathways by targeting enzymes, receptors, or bioactive lipids to consequently stimulate tumor regression and prevent metastasis. Components of altered metabolic lipid pathways that could function as potential prognostic biomarkers or therapeutic targets to prevent the growth of cancer cells or to overcome chemotherapy resistance include cholesterol [106], fatty acid synthase [114–117], autotaxin [96] ceramide [118], and CERT [108]. These and other lipids and lipid metabolism-associated biomarkers are summarized in Table 1.

**Table 1:** Classification of lipid and lipid metabolism-associated biomarkers

Biomarker	Classification	Biomarker Class	References
Cholesterol	Bioactive lipid mediator	Prognostic	[106]
Fatty acid synthase	Key metabolic enzyme	Prognostic	[114–116]
Autotaxin	LPA-producing enzyme	Prognostic	[117]
Ceramide	Bioactive lipid (metastasis-suppressor lipid)	Prognostic	[118]
Ceramide transport protein	Lipid-transfer protein	Prognostic	[108]
PI3, RGS, ADORA3, CH25H, CCDC80, PTGER3, MATK, KLRB1, CCL19, CXCL9, and CXCL10	Lipid metabolism related genes	Prognostic	[131]
Lysophosphatidic acid	Bioactive lipid mediator	Diagnostic	[120, 121]
Sulfatides	Sulfoglycolipid	Diagnostic	[122]
Phospholipase A2	Phospholipid cleaving enzyme	Diagnostic	[113, 123]
Lysophospholipids	Bioactive lipid molecule	Diagnostic	[124]
CD36	Fatty acid receptor	Diagnostic	[7]

The phospholipid composition of cancer cells as determined by lipidomic profiling could aid the differentiation between low- and high-grade tumors and malignant vs. benign cells [119]. Potential lipid ovarian cancer diagnostic markers include lysophosphatidic acid [117, 118], sulfatides [122], phospholipase A2 [113, 123], and lysophospholipids [124]. The results from a nested case-control study within the Prostate, Lung, Colorectal, and Ovarian (PLCO) Cancer Screening Trial using tandem mass spectrometry analysis of serum indicated that five arachidonic acid/linoleic acid/alpha-linoleic acid metabolites were positively associated with ovarian cancer risk: 8-HETE, 12,13-DHOME, 13-HODE, 9-HODE, and 9,12,13-THOME [125].

Ovarian cancer cells co-cultured with primary human omental adipocytes have a high expression level of the fatty acid receptor CD36 in the plasma membrane, which facilitates exogenous fatty acid uptake [7]. Thus, it has been proposed that inhibiting CD36 could be an effective treatment strategy against ovarian cancer metastasis.

Using Raman scattering imaging of single living cells and mass spectrometry analysis of extracted lipids, Li et al. observed significantly increased levels of unsaturated lipids in ovarian cancer stem cells compared to non-cancer stem cells [11]. The results of their study demonstrated that since enhanced lipid unsaturation is a metabolic marker for ovarian cancer stem cells, it can function as a target for cancer stem cell-specific therapy.

Tebbe et al. demonstrated that Metformin limits the adipocyte tumor-promoting effect on ovarian cancer by inhibiting adipocyte-mediated ovarian cancer cell proliferation, migration, expression of cancer-associated genes and bio-energetic changes [126]. Acetyl-CoA carboxylase is the target of the drug 5-tetradecyloxy-2-furoic acid (TOFA), which was found to be cytotoxic to COC1 and COC1/DDP ovarian cancer cell lines and inhibit COC1/DDP cell growth in ovarian tumor xenografts [126]. However, Pouyafar et al. demonstrated that TOFA-induced lipolysis inhibition is not as effective as glycolysis inhibition in preventing ovarian cancer stem cell differentiation into endothelial-like cells [13].

Anti-cancer drugs that perturb the cholesterol content of cell membranes can be employed to hinder lipid raft-dependent cell survival or cell death pathways. Methyl-ß-cyclodextrin (MCD) is an example of one such drug that depletes the cholesterol content of cell membranes and inhibits ovarian cancer growth while avoiding acute systemic cytotoxicity [127]. MCD has been shown to increase the efficacy of the estrogen-modulating anti-cancer drug, tamoxifen [128].

As an example of taking advantage of cancer cells’ increased dependence on saturated fatty acids, the delivery of drugs to tumors has been enhanced by loading drugs into liposomes enriched in saturated phosphatidylcholine [129]. Among the pharmaceutical benefits of liposomes are their low toxicity and ability to improve the biopharmaceutical features and therapeutic index of drugs, consequently increasing efficacy and reducing side effects [130].

Lipids also have roles as prognostic ovarian cancer biomarkers. Zheng et al. recently developed and validated an 11-gene prognostic model for serous ovarian carcinomas based on a lipid metabolism expression profile [131]. Using RNA-seq data from The Cancer Genome Atlas and Gene Expression Omnibus databases, a multi-gene prognosis model was established which consists of the following lipid metabolism-related genes: PI3, RGS, ADORA3, CH25H, CCDC80, PTGER3, MATK, KLRB1, CCL19, CXCL9, and CXCL10. This model could be utilized as a novel approach for a molecular diagnostic test to assess the prognosis and potential risk factors for patients with ovarian cancer.

## FUTURE DIRECTIONS

Ovarian cancer and its predominant subtype, high grade serous ovarian cancer, present a significant cancer burden globally. As with many other types of cancer, patients with ovarian cancer have a lower likelihood of disease-free survival if they are diagnosed with advanced disease. Although the survival rates for several forms of solid tumors have improved over the last 30 years, survival rates for patients with ovarian cancer have not changed substantially in this period. Therefore, it is of critical importance to identify new ways to diagnose and treat ovarian cancer to improve outcomes.

Like many other malignancies, ovarian cancer proliferation appears to benefit from increased levels of lipids not only in the tumor microenvironment but also in the transformed cells themselves, making lipid metabolism an attractive target for therapy. Understanding how lipid accumulation benefits the initiation and growth of ovarian cancer cells will help in determining new molecular pathways critical to tumorigenesis.

In this review, we presented evidence from the literature supporting the case that lipid metabolism in ovarian cancer cells is a crucial metabolic process that should be further investigated to improve treatment efficacy for patients with ovarian cancer. Interestingly, several lines of evidence have demonstrated that lipids (fatty acids, sterols, sphingolipids) are important mediators of classical oncogenic signaling, yet it remains unclear the extent to which the function of lipids in non-cancerous cells differs from non-transformed cells. Indeed, several novel drugs targeting various aspects of lipid metabolism pathways are being developed, which can provide new strategies for ovarian cancer treatment.

## Abbreviations

ACC: Acetyl CoA carboxylase
ACSL1: Acyl-CoA Synthetase Long Chain family member 1
AMPK: Adenosine monophosphate kinase
5-LOX: Arachidonate 5-Lipoxygenase
ACLY: ATP citrate lyase
BMI: Body mass index
CSCs: Cancer stem cells
CPT: Carnitine Palmitoyl Transferase
CERT: Ceramide transport protein
COX-2: Cyclooxygenase-2
DGAT1: Diacylglycerol O-Acyltransferase 1
DHOME: Dihydroxyoctadecenoic acid
FABP4: Fatty acid binding protein 4
FASN: Fatty acid synthase
HMGC: HMG-CoA carboxylase
HMGCR: HMG-CoA reductase
HETE: Hydroxyeicosatetraenoic acid
HODE: Hydroxyoctadecadienoic acid
THOME: Trihydroxyoctadecenoic acid
LDLR: Low density lipoprotein receptor
MCD: Methyl-ß-cyclodextrin
MIEF2: Mitochondrial elongation factor
MAGL: Monoacylglycerol lipase
OC: Ovarian cancer
ELOVL6: Fatty acid elongase 6
PGE2: Prostaglandin E2
PTEN: Phosphatase and tensin
Rb1: Retinoblastoma protein
SCD1: Stearoyl-CoA desaturase
SREBP1 and 2: Sterol response element binding protein 1 and 2
TP53: Tumor protein 53
KRAS: Kirsten Rat Sarcoma Viral Oncogene Homolog
HGSOC: High grade serous ovarian cancer
